# Podiatry as a career in the UK - what attracts Generation Z? A qualitative exploration with university and college students

**DOI:** 10.1186/s13047-021-00470-y

**Published:** 2021-04-16

**Authors:** D. Whitham, S. Whitham, M. Trowell, S. Otter

**Affiliations:** 1 School of Health Sciences, University of Brighton, 49 Darley Rd, Eastbourne, BN20 7UR UK; 2 Gills Farm, London Rd, Battle, London, UK; 3 Widening Participation Team, University of Brighton, Trevin Towers, Gaudick Road, Eastbourne, BN20 7SP UK; 4 Centre for Regenerative Medicine and Devices, University of Brighton, Lewes Road, Brighton, BN2 4AT UK

**Keywords:** Podiatry, Generation Z, Career

## Abstract

**Background:**

Training for a career in podiatry is reported to provide graduates with excellent employability, alongside professional autonomy and suitable renumeration. Yet, there has been an ongoing decline in the number of those applying to study the subject. There is limited literature associated with this topic and we sought to explore the factors that attract ‘generation Z’ (those born 1995–2010) to a potential career in podiatry**.**

**Method:**

A qualitative design framework underpinned by phenomenological principles used four focus groups over a two-year period to generate data from participants at University and in Further Education. Focus group conversations were led by external facilitator, recorded, independently transcribed verbatim and anonymised prior to thematic analysis. This was followed by external, independent verification of themes.

**Results:**

Four main themes were determined from the analysis i) a lack of awareness of podiatry; ii) podiatry: accessible course, accessible career; iii) career status; iv) breadth/opportunity of the scope of practice. Both positive and negative experiences were reported and highlighted key gaps in how the attractiveness of a career in podiatry is portrayed.

**Conclusion:**

The chronic lack of awareness of podiatry as a career clearly needs to be addressed, ideally with more positive role modelling in mainstream and popular media. The career status offered together with the breadth of, and opportunity associated with, the scope of practice should continue to be celebrated.

**Supplementary Information:**

The online version contains supplementary material available at 10.1186/s13047-021-00470-y.

## Background

Podiatry has been described as a career which offers excellent employability, provides autonomy for practitioners with good career progression opportunities together with a high earning potential [1]. However, over the past decade there has been a steady decline in the number of podiatrists – particularly those working in the NHS [2]. This trend has become more pronounced in recent years with further reductions in the numbers choosing to study the subject. In the UK, 2016–17 podiatry University Central Admissions System (UCAS) applications were down 5%, furthermore, in 2017–18 participants applying to study podiatry reduced again by 12% [3]. The loss of the means tested bursaries in 2017 was thought to be a key factor in the continuing fall in podiatry student numbers [4]. Consequently, the total number of students accepting a place to study podiatry fell by 40% between 2016–17 and 2019–20 [5]. Simultaneously, there is an increasing demand for high quality foot care services to support an aging population with increasing health and social care needs, particularly for key areas such as diabetes, falls prevention and musculoskeletal conditions [6–8]. Moreover, there seems to be an increasing public awareness of the importance of good foot health [9]. A recent Health Education England report predicted an increase in demand for podiatry of 9% by 2025 to meet core NHS service requirements, rising to 19% when related services (such as falls prevention) are considered [10]. Taking the increasing need and career opportunities together it is unclear why application rates continue to fall. This research explores what might attract young people, specifically those born between 1995 and 2010 referred to as Generation Z [11–13]. to a career in podiatry.

## Methods

### Study design

A qualitative design framework utilising underpinning interpretative phenomenological principles [14, 15], were selected. We aimed to to produce in-depth and illustrative information to identify *what* our participants perceived about podiatry and *how* this may impact their career choices [16, 17]. Influenced by the writing of Heidegger [18] we sought to interpret written narratives in relation to the individual to illuminate our underlying understanding of why participants seek podiatry as their career. This study was reported in accordance with the *COREQ* recommendations [19].

### Ethical considerations & reflexivity

The University of Brighton School Research Ethics and Governance Panel granted ethical approval. At the start of this project, two members of the research team (SO, DW) were members of academic staff and recognise these roles may influence our analysis of the data and the recommendations made. We took steps to utilise independent researchers to mitigate potential bias in the analysis of data and confirmatory meetings while ensuring confidentiality and anonymity was maintained for participants.

### Participants and settings

Two different groups of participants were sought, i) college students prior to selecting university applications that would facilitate an application to podiatry degree courses and ii) current university students already studying podiatry. College students undertaking appropriate courses (e.g., Health and Social Care or Sports & Therapy) were contacted by University outreach staff (MT) working with local college tutors who provided information sheets to potential participants. College participants did not have to demonstrate a specific interest in podiatry as previous work has suggested most college participants have limited knowledge of higher education study and potential careers [20]. College participants were able to opt in to focus group sessions held during University ‘taster’ days to maximise convenience for participants. University students already studying podiatry were recruited via email by administrative staff. Academic staff were not involved in recruitment, so potential participants did not feel coerced into taking part. Focus groups for were conducted in Universities based in the South of England and designed not to interfere with academic timetables. All potential participants were provided with information sheets and replied if they wished to opt into the study. Inclusion and exclusion criteria are detailed in Table 1. Prior to the start of each of the focus groups, participants were offered refreshments and provided informed, written consent having had the opportunity to ask further questions.

**Table 1:** Inclusion/exclusion criteria

University students	College students
Inclusion criteria Currently studying BSc (Hons) Podiatry course in their first or second year of the programme Aged 17–23	Inclusion criteria Aged 16 to 21 Undertaking A levels, BTEC, Access to Health & Social Care, International baccalaureate (IB), or other applicable courses generally applicable to University entry criteria
Exclusion criteria Age over 23	Exclusion criteria Age over 21 Aged under 16 or those unable to provide informed consent Unable to communicate competently in English to participate in a focus group

### Generation of data

Data were generated from focus group discussions with participants based on the criteria proposed by Breen et al. [21]. Focus groups were preferred as they enable participants to share and compare their experiences and to generate and develop ideas associated with those factors that would potentially attract them to podiatry [22]. Focus groups also offered the advantage of a social environment in which to articulate ideas, giving new and deeper insight into participants’ views and expectations [16]. Each focus group lasted approximately one hour. Focus group schedules (described below) were developed by the research team with questions and prompts designed to elicit a rich, thick textual description of the reasons young people might be attracted to careers in podiatry.

### Design of focus group schedules

Questions for the focus groups were derived from informal discussions with current podiatry students aged < 23 in their final year (*n* = 6). The research team were interested in:

- their reasons for choosing podiatry
- what attracted them to podiatry as a career
- If they knew any podiatrists or had received podiatry care

In addition, previous work [23] suggested the on-going reduction of applicants to podiatry might suggest an overall lower profile of the podiatry profession and its attendant opportunities together with some confusion about the role of podiatrists. Therefore, we also sought information around two further areas:

- If traditional media (e.g., television, radio) influence their decision and/or if social media had an influence on their choice or career
- Their understanding of the role of podiatry & if those expectations had been met

These ideas were subsequently developed by the research team and focus group prompts/questions (Additional file 1: Appendix 1) designed to elicit a textual description of the reasons why young people might choose podiatry as a career.

### Focus group management

For each focus group the discussion was facilitated by an independent facilitator (SW); external to the University who was experienced in this role and followed a recommended format [24, 25]:

- A welcome by the facilitator (including an opportunity to ask any questions about the study and ensuring consent was obtained for all participants)
- An overview of the topic
- A statement of the ground rules of the focus group, and assurance of confidentiality/anonymity were provided beforehand
- Questions as per the focus group schedule
- Participants had the opportunity to ask questions at the end of their session

Each group discussion was digitally recorded and transcribed verbatim and participants names were noted by the facilitator in the chronological order in which they first spoke. The raw audio data files were transcribed, and participants then anonymised by the facilitator (SW). The voice of the facilitator was transcribed in red throughout for continuity and ease of recognition and assigned the denomination ‘M’ (moderator).

### Data analysis

To reduce potential bias, data analysis was performed independently by two members of the research team (SO & DW) according to concepts outlined by Braun and Clarke [26]. Briefly, all transcripts were read several times before beginning data extraction to ensure that the researchers had a full appreciation of all of the concepts that were emerging and an overall picture of the data. In order to maintain confidentiality, audio files were not listened to as this may identify participants. Data were extracted as both single words (with the context put in brackets where needed) and as whole phrases of text to demonstrate potential themes. Initially the researchers discussed and agreed broad categories and combined into one list. This list was shared with the focus group facilitator (SW) to ensure dependability. Dependability of the analysis was then further addressed by independent verification of themes (MT). Respondent validation was not possible, as some participants had left the institution in which they were studying. Furthermore, this approach could compromise anonymity. However, to enhance the confirmability of data a further focus group was held with those studying podiatry at a different higher education institution where the themes that had been elicited were discussed and broad agreement reached.

## Results

Four focus groups were carried out, two with College participants and two with University participants with a further group discussion to confirm results as detailed above.. Throughout, educational institutions were deliberately not identified at the behest of the ethics committee and to ensure anonymity is maintained. In total 38 participants took part in the four focus groups over a period of approximately 18 months (Oct 2018 to Feb 2020) to allow for more than one cohort of participants to be involved. Sixteen university participants (12 female, 4 female) and 22 college participants (20 female and 2 male) took part. To preserve anonymity/confidentiality, participants were not directly asked socio-economic details. Independently of the research team, the recruitment team used College/University databases to identify that both groups of participants represented POLAR^1^ quintiles 1–5, together with two International students in the University cohort. POLAR (Participation of Local Areas) is a UK-wide measure to classify areas according to the participation rate of 18–19 year olds’ in higher education – those with the lowest participation rates are designated ‘quintile 1′ whereas the top 20% are quintile 5. From the analysis four main themes were determined:

1. a lack of awareness of podiatry
2. podiatry: accessible course, accessible career
3. career status
4. breadth/opportunity of the scope of practice

Each theme will be discussed in turn and supported by quotations from participants. The focus group number is indicated (FG1 = focus group one) together with what type of education institution the participant attends and the letter assigned to each participant in their relevant focus group.

### Theme 1 - a lack of awareness of podiatry

A general lack of awareness of podiatry as a profession was the most frequently discussed topic. At several points, both groups of participants demonstrated agreement that there was a lack of awareness about what podiatry was in terms of a career. For University participants in particular, lack of awareness had delayed their decision in ultimately choosing to study podiatry. Not knowing what podiatry was, or what the job role consisted of, was a common experience for both groups of participants.

*‘I didn’t know about podiatry before results day, because before results day I was going to do medicine*. I *wasn’t given enough information about it to even consider it [podiatry]’*

FG3 University participant D

*“I did not know that there was something called ‘podiatry’*”

FG1 College participant L

The only exception to this theme occurred where participants had seen a podiatrist themselves’ (*n* = 3) or had a family member treated by a podiatrist (*n* = 2). University participants also highlighted a lack of third party understanding from a wide range of sources, both personal and professional (including family members, careers advisors, peers or the general public), which contributed an overall lack of awareness.

*“It’s like a common problem, you can ask anyone, and they don’t know what it is. They say, ‘what is podiatry?’ Are you a paediatrician? Are you a foot doctor?’”*

FG2 University participant B

*“Yea, I don’t think it’s spoken about enough for us to really go out to it. Like you don’t go to college and talk about podiatry. It would just be sport science of medicine, but it won’t be something like podiatry and feet or whatever …*

FG1, College participant B

Notably, where a career preference was expressed by participants, none indicated podiatry was their first choice of course. Those who stated a healthcare career option indicated a wide range of other options (Table 2); that had (or were currently) being considered by both University and College participants.

**Table 2:** Healthcare careers that had (or were) being actively considered

Career option	Number selecting as first choice	Career option	Number selecting as first choice
Counsellor	1	Occupational therapy	1
Dietetics/Nutrition	3	Paramedic	2
Medicine	1	Pharmacist	1
Midwifery	2	Physiotherapy	8
Nursing	4	Social work	1

When exploring the reasons for the lack of awareness about podiatry, participants clearly reported podiatry had a poor overall presence, particularly in the on-line arena. In contrast other professions, particularly some in Table 2, were felt to be much more widely understood. In fact, University participants reported using overseas websites (especially from Australia, New Zealand and the USA) to gain a better understanding of podiatry as a career offered, owing to a reported paucity of high-quality information being offered in the UK.

*“The advocacy for this course [podiatry] is way bigger in countries like America, because that’s where I looked and that’s where I got the surgery element from; I never thought there was anything like that here [UK]”*

FG2University participant K

University participants were vocal in finding UK University websites of limited value in terms of their depiction of podiatry either as a course or as a career per se, as two podiatry participants commented:

*“I went to watch [refers to video] literally again the scope [of podiatry] was very narrow, just like in the XXXX Universities description itself, it’s nothing in comparison to what the course actually is”*

*FG2* University participant*, C*

*“Descriptions online for certain Universities definitely don’t do the course justice because they only give just sort of a summary, even shorter than that”*

FG2 University participant E

Continuing to explore the lack of awareness and understanding about podiatry, it was evident that participants found limitations on both popular and social media. Some University participants had viewed YouTube™ videos and reported the role of the podiatrist was portrayed as narrow and basic. One placed this in sharp focus:

*“Watching the YouTube video to see what podiatry is, and I you know, looked the description of the course on the Uni website … and when I was volunteering at XXXX hospital, I asked the podiatrist like ‘is this what you do?’ because it didn’t make sense, because it looked so basic* [on the video] *compared to what we now do on the course”*

FG2 University participant F

The lack of breadth regarding the scope of practice associated with podiatry depicted on-line, even in Institutions with podiatry courses in their portfolio, had clearly surprised many current University participants:

*“The only reason I saw podiatry was because I was trolling through like the health sciences course and I saw it and was like, ‘what the hell’, clicked on it, obviously had research, but even from the universities like own description of the course, it’s nothing in comparison to what the course actually is. The scope of practice* [of podiatry] *is huge, and is a lot bigger than what they* [Universities] *portray”*

FG2 University participant C

Equally, college participants reported their careers awareness to date had been heavily influenced by both social and popular media. This was particularly apparent where there was content that provided details regarding the role of different health professions.

*“Social media like influenced me, because obviously I want to be a midwife and, and before I wanted to do that, I was like watching this blogger person having a hypno-birth”*

FG1 College participant F

*“One born every minute definitely, that’s the best programme ever!”*

FG1 College participant E

The lack of exposure of podiatry on popular media was a particular frustration shared by many University participants

*“The only reason why doctors and lawyers are glamorised is because of movies; Grey’s Anatomy, House, you know, oh err, Liar Liar, all of these professions were in movies, podiatry and that isn’t, and I think that’s why it is not as popular”*

FG3 University participant B

The lack of exposure for podiatry was felt to be particularly true in social media, the feelings across focus groups were exemplified up by one participant:

*“ … it* [podiatry] *isn’t glamorised, … .. it isn’t published across social media, … .. it isn’t spoken about as physio is … .”*

FG2 University participant A

Moreover, the perceived attention better known professions received was a source of some frustration and led to animated conversations, particularly between University participants:

FG3 University participant E*: “I feel like everywhere it’s just like physio, physio; whereas there’s not a lot of podiatry”*

FG3 University participant H: *“There needs to be more promotion”*

FG3 University participant B *“It’s like a common problem, you can ask anyone and they … “*

FG3 University participant C *“ … they don’t know what it is”*

FG3 University participant B *“They say ‘what is podiatry?’*

FG3 University participant C *“Are you a paediatrician? Are you a foot doctor?”*

FG3 University participant B *“Or you start with the older version, Chiropodist, ‘oh yeah, chiropodist’. Especially the older people, I work in a care home and they always say ‘podiatry?’ And I say ‘chiropodist’ and they say ‘oh yeah’”*

When selecting career options, participants were keenly aware of the issue of timing associated with promoting awareness of health careers – especially for smaller health professions that were less well understood. A split in opinion was noted with some suggesting they were asked to choose careers options when they were too young to make an informed choice.

*“I feel like I didn’t know enough about university in general, let alone that much about a course I wanted to do … ”* FG3University participant E

Others, especially College participants, felt they were simply not asked about careers choices early enough.

*“At this point we are in like our third year of college; we’ve got our mind set on sort of what we want, but if it’s shown to people slightly earlier, like first year or even year 11 before they choose their courses and stuff then it would appeal more”*

FG1College participant B

*“The whole health area isn’t highlighted enough in school. You finish school and they’re like – do you want to do English, Science or Maths? It’s like if you go into science you don’t realise the wide option of everything”*

FG1 College participant F

### Theme 2 – podiatry: accessible course, accessible career

The theme of accessibility emerged from deeper considerations around awareness of podiatry and what a career in podiatry might offer. Exposure to podiatry as a profession often came late, sometimes after participants had already chosen an alternative, more popular path, such as those identified in table two. Consequently, podiatry was often seen as a second option or even as a last resort. Equally, there was a perception from several University participants that podiatry was an easier, more accessible programme to enter than more popular courses, in part because of the lack of awareness outlined previously.

*“I applied for physio, and at that time I didn’t get in”*

FG2 University participant E

*“I rejected each and every single offer that I got and then applied to XXXX* [for a different course] *and they were like, ‘Nah, sorry, but podiatry might want you?’”*

FG2 University participant F

*“ … since I had no other options, I thought why not do it?.... “I’m not going to lie, when I was applying to do this course, I was telling myself ‘Oh it’s going to be so easy’”*

FG2 University participant G

*“I was originally going to become a nurse, went through all the nursing interviews and everything and didn’t get in”*

FG3 University participant B

However, once exposed to podiatry, participants’ perceptions typically became more positive. It became clear that university participants could see a range of opportunities offered by a podiatry career and their aspirations were typically shared by college participants: even if the latter were less clear about what the podiatry profession might be able to offer in terms of career development.

The theme of accessibility naturally developed away from simply accessibility to a course of their choosing and towards the accessibility of a career that offered greater lifestyle options and a perceived greater level of control over working hours as part of participants’ career aspirations. The concept of a flexible career that allowed participants to access their lifestyle choices rather than driving their lifestyle was popular:

*“I think that the freedom to be able to you know think right, I’m a woman who is eventually going to want kids, I want a job that will, you know, earn me enough money but will also you know give me enough time to enjoy my family, enjoy my personal life and not just be so career orientated”*

FG3 University participant B

*“I think just like the flexibility of podiatry, being able to do like loads of different things … so you can go form one thing to another if you like. Let’s say if you’re bored of one aspect of it [podiatry], you can re-train in another. I think it’s just a lot of flexibility”.*

FG2 University participant L

*“I didn’t want to work odd times, just wanted set times, what time I start in the morning and able to finish at a certain time and go home”*

FG1 College participant Q

Participants were particularly attracted by being able to access control over aspects of their lifestyle that included flexible hours, something allied health careers often offer, whilst enabling the highest quality care:

*“ … because there’s a lot more control that you have, in comparison to nursing, in [podiatry] you are the consultant … I don’t want to have a boss telling me what to do all the time. I want to make the right decision for my patients without having someone else butting in”*

FG3 University participant B

### Theme 3 career status

Having identified that allied health professions such as podiatry offered an accessible career that they could fit around their lifestyle choices (as different from participants’ perceptions of medicine or nursing), a theme around ‘career status’ emerged. With greater insight into the podiatry profession (either as part of a taster day or studying on a podiatry course), all participants could see allied health careers as attractive, but for different reasons. For those already at University the wide scope of practice and clear relationship with other professions related to health (medicine, surgery and biomechanics) was a key determinant. These links appeared to make podiatry even more popular than their initial thoughts around alternative career choices:

*“I think the most appealing thing with podiatry is that it was still a medical profession … loads of different avenues you could go down because a lot of people don’t know that there’s like sports podiatry and things like that, which I think would change loads of people’s minds from physio”*

FG2, University participant C

*“ … it’s kind of similar to medicine which is why I picked it. It’s just a different route for the same thing”*

FG3 University participant D

Even without such an in-depth knowledge of the subject, those at College were attracted to aspects of the podiatry profession that hitherto had been poorly recognised:

*“ … like a lot of other job roles link to it which I didn’t know, I like to do with nutrition, so it links up. People with eating problems have feet problems so it links quite well.”*

FG4 College participant J

*“I learnt more, like I thought it was just feet but then looking around clinics and stuff it’s shown me how broad the range pf stuff they actually do is, like especially the medicines and stuff”*

FG1 College participant B

Participants from each focus group were in general agreement that their ideal career needed to be able to offer good prospects post-qualification. Those at University were quick to recognise the shortage of podiatrists and valued the opportunities this might offer them.

*“ … job security, you’ll walk out and you’re good, you’ll walk into a job and then you can progress. It’s such a niche thing that there are only going to be so many people coming out that year with you and there’s obviously a stupid amount of jobs, so you’ll be able to go into that job process very, very quickly.*

FG2 University participant C

*“There is a need for podiatrists and you are quite likely to get a job once you graduate. It’s not as competitive as other professions”*

FG3 University participant D

*“This [podiatry] definitely gives you something, because every year there’s a bunch of third years who have already secured jobs for when they graduate, so it definitely pays off”*

FG2 University participant J

In addition, the range of opportunities for career progression, the perceived speed of promotion was also reported to be a key advantage by those already attending University.

*“The progression of the career as well. You’re not, you don’t just graduate and then have to wait five or six years before you can move up the pecking order”*

FG2 University participant C

It should be noted however, that it was still important to participants that the individual practitioner remained in control of their own development:

*“ … the possibility that it [podiatry] can develop further, like it’s how far YOU want to take it, you know. You’re very much in control of it. You’re in control of you patients you’re in control of your work hours, you’re in control of you know, how far you want to go with it and I think that’s the good part about it, there’s so much control in so many different aspects.”*

*FG3 University participant B*

In parallel with being having good job prospects, there was a sense of being successful and the suggestion that podiatry was seen by some as a potentially lucrative career. For University participants the salary had been identified and was understood prior to their course commencing.

*“I don’t see why people focus so much on ‘oh crap I am going to have to pay this off’ I’m like ‘yeah but you are not going to be working in a minimal career on minimum wage’”*

FG2 University participant C

*“ … he was saying how much he was earning, and I was like ‘Ah wow’”*

FG2 University participant E

### Theme 4 breadth/opportunity for scope of practice

Rather than solely focus on financial remuneration and career status, many participants were more mindful of the breadth of practice a podiatry career might offer them. In particular the links to medicine and surgery, even if tied to the need for extensive further study, was an area University participants returned to, particularly where is set them apart as podiatrists from other professions:

*“It’s quite invasive, it’s very similar to medicine in a sense because you do surgery, you do all these other things that you wouldn’t do as a physiotherapist, that’s what I liked in a sense.”*

FG2 University participant I

*“I saw that there was a link between podiatry and biomechanics”*

FG3 University participant E

*“ … the* [podiatric] *surgery is what appealed to me most”*

FG3 University participant D

Taken together, the wide scope of practice associated with podiatry, together with a perceived ability to navigate through the career structure in a flexible manner linking with other specialisms gave participants a sense of responsibility which came hand-in-hand with the strong ethos of caring exhibited throughout the focus groups. University participants were quick to identify how important this aspect of the podiatry profession was to them:

*“ … helping people and facilitating people in their daily lives, I think that’s quite a big point on this course”*

FG2 University participant K

*“There’s also like the satisfaction of knowing that you made someone feel better.”*

FG2 University participant G

For some participants, the opportunity to build professional relationships with their patients over a period of time was a unique aspect to podiatry and offered an opportunity to set podiatry apart from other healthcare careers:

*“You don’t go into a health profession if you don’t care and what I think pushes podiatry out and is different from the rest of them is … you have that constant contact with your patient, you just don’t see them and then they’re gone”*

FG2 University participant C

The sense of helping people was equally attractive to college participants, who, even if they were not as aware of the breadth and scope of podiatry practice in the same way as University participants, did identify with a need for, and importance of, a caring profession:

*“I just know that I want my job to be in health and social care just to help people”*

FG4 College participant L

*“ … to do something that could change someone’s life”*

FG1 College participant L

*“Yeah, I do like being able to sort of help someone”*

FG1 College participant C

The practical nature of podiatry aligned closely with the idea of actively helping people was particularly attractive to many college participants. The breadth of ‘hands on’ activities they had experienced had highlighted in their taster day heightened the importance of a practical, experiential career to them

*“I want to be very hands-on”* [Loud agreement from peers]

FG4 College participant C

*“Yeah practical, not sitting down, practical”*

FG4 College participant E

## Discussion

To the best of our knowledge this is the first work to explore what Generation Z students feel about, and want from, the profession of podiatry. Our results brought into sharp focus the number of participants who were largely unaware of podiatry as a profession, whether they were thinking about their career options, or for those studying podiatry and reflecting on their journey to their career choice. It is perhaps not surprising therefore that applications to podiatry courses have been gradually falling, the loss of the NHS bursary in 2017 simply accelerating this pattern. Podiatry courses across the south of England have seen a greater decline in student numbers [27], leading to widespread fears about course viability. At the time of writing there were 120 public sector vacancies for podiatrists across the UK [28]. Moreover, these data must be set in the context of a decade of decline, which has seen a 12% reduction in the numbers of podiatrists working in the NHS between 2010 and 2019 [5]. The shortage of podiatrists and overall lack of understanding of the profession has also become part of a more national conversation as reflected in national newspaper reports [29, 30].

The lack of understanding about the podiatry profession seems to be underpinned by a complex interrelated set of beliefs. Our participants noted a lack of positive role modelling in both popular and social media outlets, which was often in contrast to that of many other professions who benefit from more positive depictions on traditional media outlets via a plethora of programmes both documentary and dramatic. The impact of such demonstrations should not be underestimated. Recent work highlighted that 47% of children aged 10–16 turn to YouTube™ for careers advice rather than going to teachers or parents. In addition, 37% of young people turned to celebrities and ‘influencers’ as their first choice for careers advice [31]. The lack of positive role models in podiatry appeared to be compounded by a worryingly limited view of the scope of practice of the profession as outlined by some on-line sources, including Higher Education Institutions offering podiatry courses.

The experiences of Allied Health Professions evidenced in recent publications [32–35] are key to positive role modelling within the profession and need to be disseminated more widely. The work of health professionals is currently held in high esteem by the public and reportedly led to a surge in applications to healthcare courses [36–38]. Such experiences also fit the broader concept of role modelling where positive role models in healthcare have been seen as important to the professional development of both undergraduate and junior clinicians [39, 40]. Positive role modelling has been successfully used in the Higher Education setting; for example, student ambassadors at University outreach events. Prospective students, particularly from under-represented groups, are more likely to identify with positive role models, which in turn increases their understanding of higher education study, student life and helps support their decision making.

Positive role modelling could be particularly important for widening participation candidates who encounter a range of barriers to participating in Higher Education including financial limitations, transport, diversity and assessment methods, all of which can lead to a sense of not fitting in at university [41]. The use of role models to encourage participation for under-represented groups appears to be a key and widespread component of interventions in some sectors. For example, a study of medical students highlighted the importance of role modelling and suggested that widening participation programmes needed to choose positive role models to intervene early [42]. A further analysis of Higher Education students working as student ambassadors provided prospective applicants with a role model from which to develop more accurate perceptions of higher education and challenge negative stereotypes. Furthermore, this relationship with student ambassadors were reported to be key to enhancing confidence, motivation and attitudes towards Higher Education [43].

The positive benefits offered by a career in podiatry, notably professional autonomy, financial reward, broad scope of practice, career versatility and involvement in a caring profession [23, 44], were well understood by those studying podiatry. Many wider components, particularly autonomy, flexibility and a sense of helping were equally attractive to college students. These core elements of the podiatry profession are accurately reflected in some widely available careers information [45, 46]. However, other publications offer more limited information and did not fully reflect our participants’ perceptions [47]. Our findings are not unique to podiatry as other professions have reported an under-representation of both the role and impact of their profession in communications with the wider public [48, 49]. Equally, we report a worrying disconnect between some of the information available about podiatry from both careers’ services and university websites with what younger people are looking for from a career. In the ‘worst-case scenario’ those who may be attracted to a podiatry career may not actively seek the profession owing to a lack of knowledge about the profession. Then, when/if information is sought, this may not fully reflect the determinants prospective applicants find most attractive. Interestingly, financial reward, which might be thought to be important, was rarely mentioned in our focus groups, unless it was specifically brought up by the facilitator. Yet this information is often at the forefront of many careers’ websites. This finding highlights the need for careers information, advice and guidance in schools and colleges to be holistic and to appeal to what our participants self-identified was important to them. In this regard it is important to match the depth knowledge associated with podiatry and the diverse range of specialisms within the field with what potential applicants want to know. Equally, the overlap and links with other courses such as Medicine, Sport Science and Biology, may not be obvious to potential participants and needs to be signposted in online resources. Provision of information, advice and guidance to participants at secondary school is a low-cost, light-touch opportunity. The more promising interventions are those that are tailored to the participants, start early and are integrated with other forms of support, such as broader careers advice and guidance [50].

Recently, professional, regulatory and statutory bodies have combined with higher education providers to identify and implement mechanisms to reverse the decline in applications, particularly for smaller allied health professions such as podiatry. They have introduced a range of strategic interventions, for example the Strategic Interventions in Health Education Disciplines initiative [51]. Our work highlights how important these initiatives will continue to be in reversing the decline in numbers applying to study podiatry. Ongoing work around career opportunities remains urgently needed with education providers at all levels - school, post-16, and university. Timely information, advice and guidance for participants, parents/carers are key, particularly where this can be delivered through on-campus tasters, events and talks. Early interventions through widening participation activities can potentially shape later outcomes [52], and strategies such as subject tasters are also important for showcasing different careers that participants may not have encountered in their school curriculum. A range of activities (taster days, Saturday clubs and summer schools) can provide secondary school and college students with valuable information and advice around future career choices. In addition to these interventions, continuing professional development for school and college staff, to ensure up-to-date information is available and effectively disseminated to an increasingly technologically capable generation is important.

The main limitation of this work is that some members of the research team were University employees and in some cases podiatrists. We acknowledge that this may potentially influence our interpretation of the results and have led us to potentially place greater emphasis on certain factors. The robust methodology employed, offers assurance of the trustworthiness of our interpretation of the data. The inclusion of more than one cohort of participants gives further confidence that we are not simply reporting a ‘one off’ event. It should be noted that recent announcements associated with support for Nursing, Midwifery and Allied Health courses via the NHS learning support fund [53] are welcome but were made after our focus groups were held.

In conclusion, there is a chronic lack of awareness of podiatry as a career, and an urgent need to draw attention to what the profession offers, ideally with more positive role modelling in mainstream and popular media. The career status offered by podiatry, together with the breadth of and opportunity associated with, its scope of practice, should continue to be celebrated.

## Supplementary Information

**Additional file 1:.**

## Data Availability

Original transcripts are available on a University of Brighton database https://researchdata.brighton.ac.uk/id/eprint/211
