# Supplementary material for: Podiatry as a career in the UK - what attracts Generation Z? A qualitative exploration with university and college students
Source: J Foot Ankle Res. 2021 Apr 16;14:33. doi: 10.1186/s13047-021-00470-y (PMC8052815; doi:10.1186/s13047-021-00470-y)
Supplement: Supplementary file 1 — Additional file 1:. [file 13047_2021_470_MOESM1_ESM.docx]

**Focus group schedule for college students**

1 What did you want to do when you started your college course?

Prompt – it is OK not to have been sure

2 Do you know any podiatrists or have received podiatry care

Prompt can be either yourself or close relatives

3 What do you want from a career?

Prompts – monetary reward, flexible work patterns, autonomy, personal satisfaction, a level of responsibility

4 What has influenced your career choice(s) so far?

Prompts (television, radio, social media etc.)

5 What was your perception of podiatry before you came today?

6 Has today changed your perception?

7 Would you be attracted to podiatry as a possible career?

8 Why might you not consider podiatry as a career option?

**Focus group schedule for University students**

1 Were you initially attracted to podiatry as a possible career?

2 When did you chose to apply to your podiatry course?

3 Do you know any podiatrists or have received podiatry care

Prompt can be either yourself or close relatives

4 What do you want from a career?

Prompts – monetary reward, flexible work patterns, autonomy, personal satisfaction, a level of responsibility

5 What influenced your career choice(s) & influences your application to podiatry?

Prompts (television, radio, social media, University websites etc)

6 What was your perception of podiatry before you started the programme?

7 Has the course so far changed your perception?

8 Why might you not consider podiatry as a career option?
